# Persistent effects of cyclic adenosine monophosphate are directly responsible for maintaining a neural network state

**DOI:** 10.1038/s41598-019-45241-9

**Published:** 2019-06-21

**Authors:** Matthew H. Perkins, Klaudiusz R. Weiss, Elizabeth C. Cropper

**Affiliations:** Icahn School of Medicine at Mt. Sinai, Department of Neuroscience and Friedman Brain Institute, One Gustave L. Levy Place, Box 1065, New York, NY 10029 USA

**Keywords:** Neural circuits, Feeding behaviour

## Abstract

Network states are often determined by modulators that alter the synaptic and cellular properties of the constituent neurons. Frequently neuromodulators act via second messengers, consequently their effects can persist. This persistence at the cellular/molecular level determines the maintenance of the state at the network level. Here we study a feeding network in *Aplysia*. In this network, persistent modulation supports the maintenance of an ingestive state, biasing the network to generate ingestive motor programs. Neuropeptides that exert cyclic adenosine monophosphate (cAMP) dependent effects play an important role in inducing the ingestive state. Most commonly, modulatory effects exerted through cAMP signaling are persistent as a consequence of PKA activation. This is not the case in the neurons we study. Instead maintenance of the network state depends on the persistence of cAMP itself. Data strongly suggest that this is a consequence of the direct activation of a cyclic nucleotide gated current.

## Introduction

Second messenger signaling is a fundamental mechanism in the modulation of neural networks. Modulation of synaptic and cellular properties is critical for establishing network states which facilitate performance of behavioral responses that are appropriate to specific contexts or recent experiences^1–3^. Cyclic adenosine monophosphate (cAMP) may be the most intensively studied second messenger. Behavioral states that have been linked to its modulatory actions include attentional states^4^, states of pain sensitization^5,6^, and states of hyperactivity^7–9^. For each of these neuromodulatory actions, cAMP signaling through a downstream effector, namely PKA, has been implicated in establishing or maintaining the associated behavioral state. In this work, we describe an atypical mechanism of persistent neuromodulation. Here cAMP activates a current that changes cellular activity, meaning the duration of modulatory action depends directly on the persistence of cAMP activity.

We study the network that mediates feeding behavior in the mollusc *Aplysia*. This network is like many others in that its activity is configured and reconfigured by modulatory neurotransmitters to generate ingestive and egestive motor programs^10^. These experiments focus on ingestive activity. Ingestive motor programs are triggered by a command-like neuron, cerebral buccal interneuron 2 (CBI-2). Interestingly, the first cycle of activity generated is not immediately ingestive. Instead, phase relationships of motor neurons are poorly defined and motor activity is referred to as having intermediate characteristics. Cycles only become ingestive with repetition, i.e., when they are repeatedly induced with a relatively short intercycle interval (ICI). This is a form of repetition priming.

When CBI-2 is stimulated so that priming is induced it releases two neuropeptides, FCAP and CP-2, which exert widespread effects on the feeding circuitry^11–17^. The combined actions of FCAP and CP-2 (FCAP + CP2) promote ingestion by progressively modifying the activity of the circuitry that controls the opening and closing of the organ *Aplysia* use to grasp food, the radula. Much previous work studied modifications of activity in the radula closer circuitry. This report focuses on radula opening, namely the B48 radula opener motor neurons.

As activity becomes ingestive there are progressive increases in the B48 firing frequency. Previous work established that these increases are primarily due to an FCAP + CP2-induced increase in excitability that persists for at least fifteen minutes^16^. This persistent state is not induced in the presence of cAMP antagonists, so it is clearly cAMP-dependent^12^. A question addressed here was: is PKA activation also required? Interestingly, we demonstrate that it is not. Instead, our data strongly suggest the persistence of cAMP itself, and the induction of a cAMP-gated inward current. Cyclic nucleotide gated (CNG) currents are present in neurons in the CNS of multiple species and are starting to receive increasing attention as potential mediators of neural plasticity^18,19^. The present findings demonstrating that induction of a cyclic nucleotide gated current can induce a persistent excitability increase, and thereby alter network state, are likely to be of broad interest.

## Results

### Priming of B48 activity does not depend on PKA

**Figure 1:**
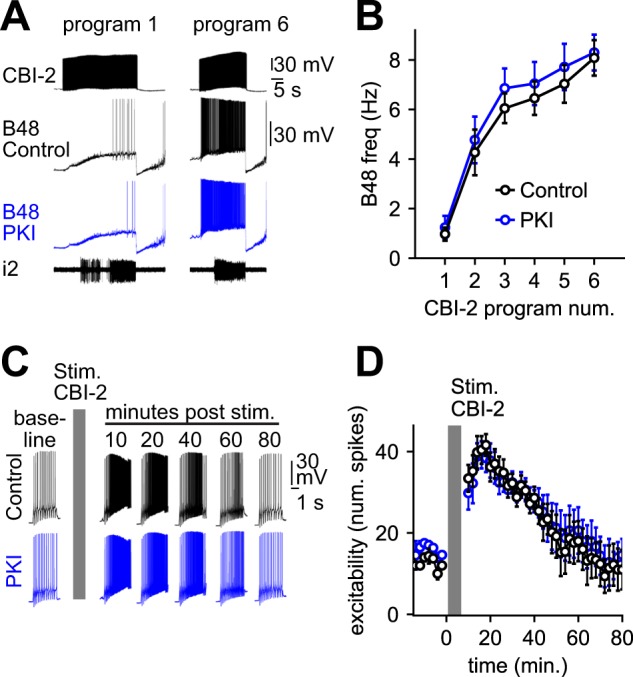
PKA is not required for the induction of ingestive priming (see also Fig. S1). (**A,B**) PKI loading does not impact priming of B48 activity observed with repeated CBI-2 stimulation. Six cycles of motor activity were triggered by CBI-2 in preparations in which pairs of B48 neurons were loaded intracellularly with vehicle (control, black) or PKI (blue). Increased B48 firing, i.e. priming, was observed in both cases. (**C,D**) CBI-2 induced increases in B48 excitability persist in the presence of PKI. B48 excitability was measured by injecting constant current pulses before priming (baseline) and for 80 min after priming in neurons injected with vehicle (control, black) and in neurons injected with PKI (blue). Gray bars indicate priming (Stim CBI-2). PKI loading had no effect. Traces are membrane voltage recorded from bilateral pairs of B48 neurons, during CBI-2 elicited motor programs (**A**) and during excitability tests (**C**). Sample sizes: Panel B (N = 5), Panel D (N = 5), where N = number of preparations.

To determine whether PKA activation is necessary for the induction of ingestive priming one of the two B48 neurons was injected with Protein Kinase Inhibitor (PKI)^20^. The other B48 neuron was loaded with vehicle. When CBI-2 was stimulated the two neurons fired at similar frequencies (F_(1,44)_ = 3.62, P = 0.064, N = 5), and in both cases the firing frequency progressively increased (Fig. 1A,B) (Vehicle: t_(4)_ = 9.97, P = 0.00057, PKI: t_(4)_ = 10.68, P = 0.00044). Since PKI had no effect we conducted ‘positive control’ experiments using pleural sensory neurons. As has been reported^20^ we found that PKI prevented serotonin induced increases in excitability (Fig. S1A,B). In vehicle loaded neurons 2.0 ± 0.32 spikes were triggered by current pulses before serotonin, and 14.4 ± 2.16 were triggered after (t_(4)_ = 6.08, P = 0.01, N = 5). In PKI loaded neurons 1.8 ± 0.2 spikes were triggered before serotonin and 3.0 ± 0.84 were triggered after (t_(4)_ = 1.63, P = 0.533, N = 5).

Although these data indicate that PKA is not necessary for the induction of priming, they do not indicate whether it is activated with a delay to maintain the ingestive state. To address this issue we determined whether CBI-2 induced changes in B48 excitability persist in PKI loaded neurons. We found that they do (Fig. 1C,D). In control neurons it took 52.4 ± 11.1 min for excitability to return to 37% of its peak level after CBI-2 stimulation. In PKI loaded cells it took 53.6 ± 10.6 minutes (t_(4)_ = 0.43, P = 0.69, N = 5). Similarly, we monitored B48 excitability after FCAP + CP2 superfusion (Fig. S1C,D). Again there was no difference between the excitability of control and PKI loaded neurons (F_(1,263)_ = 3.24, P = 0.073, N = 4). These data indicate that PKA activation is not necessary to maintain the ingestive state.

### Priming activates a persistent current in B48 that is similar to a characterized cAMP-gated current

**Figure 2:**
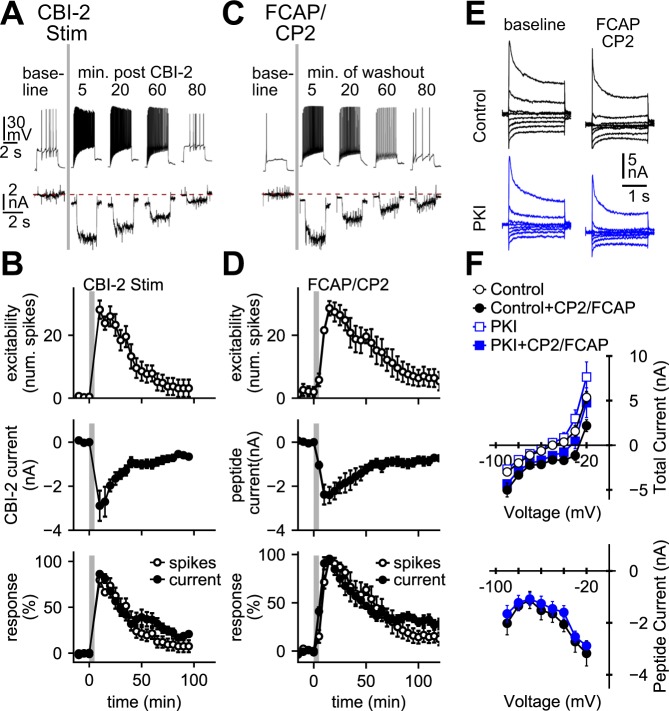
Ingestive priming induces a PKI insensitive inward current that persists and dissipates in parallel with changes in excitability. (**A,B**) CBI-2 stimulation increases B48 excitability (top traces in (**A**), top plot in (**B**), bottom plot in (**B**)), and activates an inward current (bottom traces in (**A**), middle plot in (**B**), bottom plot in (**B**)). (**C,D**) FCAP + CP2 superfusion (1 µM each) increases B48 excitability (top traces in (**C**), top plot in (**D**), bottom plot in (**D**), and activates an inward current (bottom traces in (**C**), middle plot in (**D**), bottom plot in (**D**)). In (**A–D**) excitability was measured by injecting a 3 s constant current pulse. Plotted data show currents measured during a 2 s step to −30 mV. The dotted red line in (**A**) and (**C**) marks zero current. In (**B**) and (**D**) data plotted are B48 excitability (top plot), and inward current induced (middle plot). In the bottom plot data shown above were replotted together as a percentage of the maximal response. (**E,F**) Peptides induce an inward current that is not blocked by PKI. Currents were measured during 2 s steps delivered between −90 and −20 mV in 10 mV increments from a holding potential of −60 mV. Recordings were made before (baseline) and after peptide superfusion (FCAP + CP2 1 µM each) in vehicle loaded neurons (control, black) and in PKI loaded neurons (blue). In (**F**) total currents are plotted in the top graph and difference (peptide-induced) currents in the bottom graph. Sample sizes: Panel B (N = 5), Panel D (N = 5), and Panel F (N = 4).

A current directly gated by cAMP has been characterized in molluscs^21–27^. After priming, cAMP levels could remain elevated, which could lead to persistent induction of the inward current, and persistent excitability increases. This suggests that after priming the induced current and the excitability increase should decay in parallel. We found that they do (Fig. 2A,B). For example, with voltage clamp steps to −30 mV it took the inward current 59 ± 11.5 minutes to fall to 37% of its peak value (Fig. 2B middle plot). With current clamp steps, it took 62 ± 6.44 minutes for the increased spike number (excitability) to fall to 37% of its peak value (Fig. 2B top plot). The two time constants were not significantly different (t_(4)_ = 0.35, P = 0.74, N = 5, paired) (Fig. 2B bottom plot). Similar results were obtained when the peptides FCAP + CP2 were superfused (Fig. 2C,D). With peptide superfusion it took 67 ± 9.8 and 58 ± 14 minutes for the increase in excitability and the inward current to subside, respectively. These time constants were not significantly different (t_(4)_ = 0.8, P = 0.46, N = 5, paired).

A further prediction of the model is that induction of the inward current should occur in the presence of PKI. To test this possibility we used FCAP + CP2 to activate the current. The current induced in PKI loaded cells was not different from the current in vehicle-loaded cells (Fig. 2E,F) (F_(1,76)_ = 1.64, P = 0.2, N = 4). These data indicate that a persistent current is induced in B48 that is not blocked by PKI.

**Figure 3:**
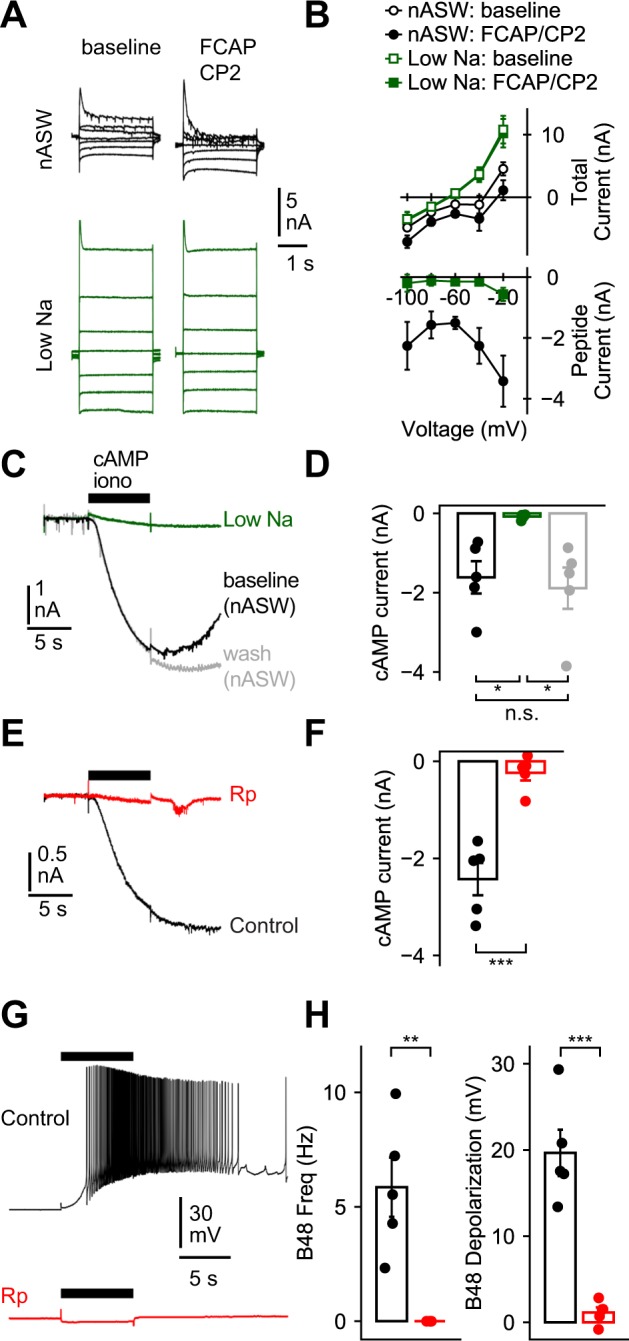
The current induced by ingestive priming is similar to a CNG sodium current (see also Fig. S2). (**A,B**) Peptide-induced currents are not observed in low sodium saline. Currents were measured during 2 s steps delivered between −100 and −20 mV in 20 mV increments from a holding potential of −60 mV. Recordings were made before (baseline) and after peptide superfusion (FCAP + CP2, 1 µM each) in normal ASW (nASW, black) and in a saline with 95% of the sodium removed (low Na, green). In (**B**) the top graph plots total currents, the bottom graph difference (peptide-induced) currents. (**C** and **D**) cAMP induced currents are not observed in low sodium saline. Currents were induced by iontophoresis of cAMP (horizontal black bar) in nASW (baseline, black), in a saline with 95% of the sodium removed (low Na, green), and after returning to nASW (wash, gray). (**E–H**) cAMP induced currents and changes in membrane potential are not observed in Rp loaded neurons. Recordings were obtained from vehicle-loaded neurons (control, black) and in neurons preloaded with Rp-cAMPS (Rp, red). (**E**) and (**F**) show currents induced by cAMP at −60 mV and (**G**) and (**H**) show changes in membrane potential at −60 mV. In (**H**) changes in firing frequency are on the left, and changes in membrane potential after spiking are on the right. Sample sizes: Panels B, D, F and H (N = 5).

The current that is directly gated by cAMP is primarily a sodium current^21,22^. To determine whether the same is true of the current in B48, we eliminated most of the sodium in the perfused ASW. This substantially reduced the FCAP + CP2 induced current (Fig. 3A,B) (F_(1,42)_ = 48.32, P < 0.0001). For example, in control cells FCAP + CP2 induced a −1.5 ± 0.2 nA current measured at −60 mV. When 95% of the sodium was replaced by N-methyl-D-glucamine (NMDG), the change in the holding current was significantly less than the current induced under control conditions, i.e., it was −0.15 ± 0.08 nA; (t_(4)_ = 7.36, P = 0.0018, N = 5). It was also not different from zero (t_(4)_ = 1.8, P = 0.15, N = 5). This is consistent with the idea that sodium is the mobile ion. Alternatively, the current could be carried by a different ion but could have been blocked by the NMDG. To explore this possibility we measured the current in ASW with only 50% of the sodium replaced with NMDG. If the current is carried by sodium, this should reduce the magnitude of the current by ~50%. If NMDG blocked the current, we would expect a larger reduction. When currents measured in normal ASW (nASW) were divided by two they were not different from currents measured in 50% sodium (Fig. S2A–D) (F_(1,104)_ = 2.42, P = 0.123, N = 6, see methods for calculation).

Another feature of the cyclic nucleotide gated current is that it is potentiated by reductions in extracellular calcium^21,22,25^. We found that the same is true for the B48 current (Fig. S2G–J (F_(1,28)_ = 26.29, P < 0.0001). For example, in nASW FCAP + CP2 induced a current of −0.85 ± 0.15 nA at a holding potential of −60 mV. In low calcium ASW the current was −3.41 ± 0.56 nA (t_(6)_ = 4.4, P = 0.004, N = 4).

If the current in B48 is gated by cAMP it should be induced by direct cAMP application. Further, the cAMP activated current should be similar to the current induced by peptides. We found that this is the case. Iontophoresis of cAMP in nASW induced a −1.61 ± 0.41 nA current, which was significantly larger than the current cAMP induced in very low sodium saline, −0.08 ± 0.03 nA (Fig. 3C,D, t_(4)_ = 3.98, P = 0.049). When only 50% of the extracellular sodium was replaced by NMDG, the cAMP induced current was not significantly different from its value measured at baseline divided by two (Fig. S2E,F) (t_(3)_ = 1.4, P = 0.23, N = 4, paired, see methods). These data indicate that the current induced by cAMP, like the peptide-induced current, is primarily carried by sodium.

Other experiments were conducted in nASW and a low calcium ASW (Fig. S2K,L). In nASW, cAMP induced a current of −1.62 ± 0.33 nA at −50 mV. In low calcium ASW, the current was significantly potentiated, i.e., it was −2.63 ± 0.35 nA, (t_(2)_ = −30.0, P = 0.003, N = 3, paired).

Finally, CNG channels have a conserved region in the cytoplasmic C-terminal domain that is highly homologous to the cyclic nucleotide-binding domain of cAMP-dependent PKA. Consequently they can be directly antagonized by the PKA inhibitor Rp-cAMP (even when effects are not PKA mediated)^28^. We found that Rp-cAMPS preloading markedly reduced the magnitude of cAMP induced currents in B48 (Fig. 3E,F). In control cells cAMP activated a current of −2.43 ± 0.33 nA at −60 mV, whereas in cells preloaded with Rp-cAMPS, the induced current was −0.24 ± 0.16 nA (t_(8)_ = 5.9, P = 0.00035, N = 5) (i.e., not different from zero; t_(4)_ = 1.5, P = 0.21, N = 5). Further, iontophoresis of cAMP into control cells under current clamp conditions caused neurons to fire at 5.85 ± 1.3 Hz and then remain depolarized by 19.7 ± 2.7 mV (Fig. 3G,H). None of the Rp preloaded cells spiked in response to cAMP or were significantly depolarized (t_(4)_ = 1.9, P = 0.13, N = 5). Taken together these data suggest that priming induces a persistent current in B48 that is similar to a previously characterized CNG current.

### Persistent activation of cAMP signaling is necessary to maintain CBI-2 priming

**Figure 4:**
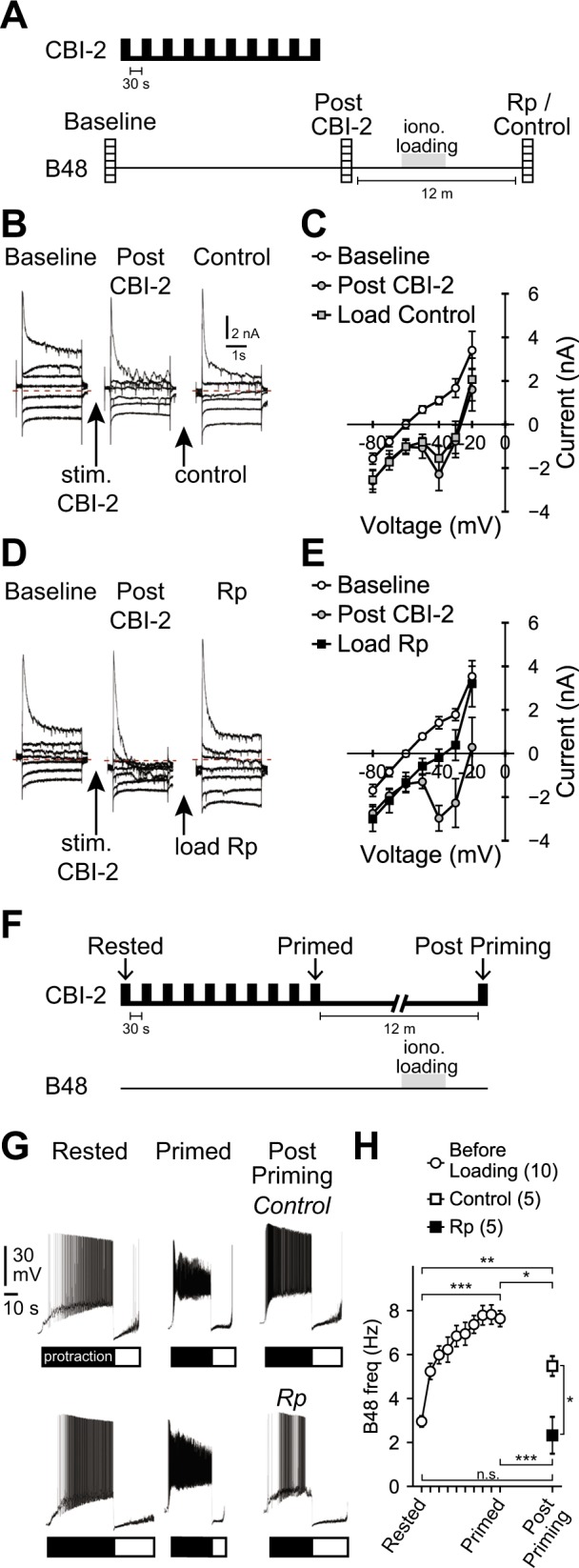
(**A**) Paradigm used in (**B–E**). Currents were measured during 2 s steps delivered between −80 and −20 mV in 10 mV increments from a holding potential of −60 mV before priming (baseline), immediately after priming (Post CBI-2), and 12 min after priming in neurons loaded with either vehicle (Control) or Rp-cAMPS (Rp). (**B–E**) Currents in Rp loaded neurons were smaller than currents in control neurons. (**F**) Paradigm used in (**G**) and (**H**). The B48 firing frequency was measured before priming (Rested), immediately after priming (Primed) and 12 min after priming in Control (vehicle loaded) neurons and in neurons loaded with Rp-cAMPS. (**G,H**) Priming is not observed at 12 min when neurons are loaded with Rp-cAMPS. (**G**) Current clamp recordings from control (vehicle loaded) and Rp-loaded (Rp) neurons. The bars under the traces indicate radula protraction (black) and radula retraction (white). Sample sizes: Panel C (N = 9), Panel E (N = 6), Panel H (N = 10, unpaired [Control = 5, Rp = 5]).

Our model postulates that the current induced in B48 persists as does priming. This suggests that blocking the current after CBI-2 has been stimulated should eliminate priming. To determine whether this is the case we measured currents immediately after priming, loaded cells with vehicle or Rp-cAMPS and then measured currents twelve minutes after priming (at a time point when persistent excitability increases are observed^16^) (Fig. 4A). Loading cells with vehicle had no effect (Fig. 4B,C). For example, before loading the inward current was −3.38 ± 0.73 nA during steps to −40 mV. After loading, it was −2.65 ± 0.53 nA (t_(8)_ = 1.27, P = 0.24, N = 9). In contrast, currents were reduced when cells were loaded with Rp-cAMPS (Fig. 4D,E). For example, before loading CBI-2 induced a current of −4.38 ± 0.69 nA during steps to −40 mV. After loading, the inward current was −1.6 ± 0.36 nA (t_(5)_ = 4.6, P = 0.011, N = 6). Importantly, the input resistance of cells loaded with Rp was not significantly different from the input resistance of control neurons (11.58 ± 2.14 MOhm for the Rp loaded cells vs. 11.43 ± 1.58 MOhm for control cells (t_(8)_ = 0.058, P = 0.95, N = 5)). These data indicate that injecting Rp-cAMPS after priming impacts current induction.

The final question we addressed was, will delayed elimination of the inward current impact the retention of the ingestive state? After Rp and vehicle loading we triggered a single cycle of a motor program and measured the B48 firing frequency to determine whether it was still above baseline levels (Fig. 4F). We found that Rp loading had a significant effect (F_(1,91)_ = 12.19, P < 0.001)) (Fig. 4G,F). The B48 firing frequency was 2.95 ± 0.26 Hz before priming and increased to 7.8 ± 0.44 Hz after priming (t_(9)_ = 14.37, P < 0.001, N = 10). Twelve minutes later the frequency was still above baseline in control neurons (5.48 ± 0.45 Hz; t_(4)_ = 6.24, P = 0.003, N = 5) but not in Rp-loaded cells (2.32 ± 0.84 Hz; (t_(4)_ = 0.82, P = 0.46, N = 5). These data suggest that persistent induction of the inward current is necessary to maintain priming.

## Discussion

Priming in B48 is cAMP dependent^12,16^, but here we show that it does not involve PKA activation. It does, however, depend on the maintained induction of an inward current. We suggest that the inward current persists because cAMP levels remain elevated and cAMP directly gates the relevant current.

Multiple mechanisms have been described that can lead to persistent increases in cAMP levels, e.g., when agonist concentrations are high, phosphodiesterase activity can be ‘overwhelmed’^29^. In other situations internalized G-protein coupled receptors (GPCRs) continue to be coupled to adenylate cyclase inside an activated cell leading to persistent cAMP production^30–32^.

Also supporting our model are data that demonstrate that the inward current activated in B48 is similar to a characterized molluscan current that is cAMP gated. Further, the B48 current, like other CNG currents, is blocked by Rp-cAMPS. Notably, Rp-cAMPS eliminates priming when it is injected after priming has occurred. These data are important because although we demonstrate that persistent effects are not mediated via PKA activation, a transient increase in cAMP could be followed by the activation of a different signaling molecule. The delayed loading data indicate that this is not likely to be the case. Rp-cAMPS generally acts by competing with cAMP binding^33^. Consequently, Rp-cAMPS is not likely to be inhibitory once cAMP is no longer present.

The model that we suggest is likely to apply in some contexts but not others. Others have shown that the GPCR internalization mechanism described above is cell type specific^35^. Further, here we show that cAMP mediated effects in *Aplysia* sensory neurons are much less persistent (Fig. S1A,B), a finding that is in agreement with earlier work that looked at the time course of the activation of *Aplysia* adenylate cyclase in a perfused membrane preparation^34^.

The current that we study is primarily activated at depolarized potentials. This makes it ideally suited for its purpose. Activation of the current induces an excitability increase that is selectively manifested during a particular phase of the feeding motor program (i.e., during protraction, which is when B48 receives excitatory synaptic input)^16^. In a previous study, the dynamic clamp method was used to test the functional significance of a current with this voltage relationship. Results showed that this current is both necessary and sufficient for the increase in B48 activity observed with CBI-2 priming^16^.

The current voltage relationship distinguishes the B48 current from canonical CNG channels^36^, and from the hyperpolarization-activated inward current (Ih), a type of current also sensitive to increases in intracellular cAMP^37^ that has recently been described in *Aplysia*^38^. Notably, results from the related mollusc *Pleurobranchaea* suggest that the current voltage relationship observed here might be specifically associated with feeding. In *Pleurobranchaea*, two different types of cAMP gated sodium currents have been described, one of which is activated by depolarization, and present in buccal ganglia, and a second that is reduced with depolarization and found in cells in pedal ganglia^21^.

In conclusion, CNG currents are present in neurons in the CNS of multiple species and are starting to receive increasing attention as potential mediators of various forms of neural plasticity^18^. The present findings demonstrating that induction of a cyclic nucleotide gated current can induce a persistent excitability increase, and thereby alter network state, are likely to be of broad interest

## Methods

*Aplysia californica* weighing between 90–400 grams were purchased from Marinus Scientific, LLC (Long Beach, CA), and maintained in an artificial seawater tank around 16 °C. Aplysia are hermaphrodites, i.e. both male and female. Animals were anesthetized by an injection of isosmotic magnesium chloride (1150 mOsm), equal to 60% of the body volume. Dissection and surgical preparation began 15 min after the animal was flaccid and non-responsive.

### General electrophysiological methods

Buccal and cerebral ganglia were prepared by desheathing in a 1:1 v/v mix of nASW and isosmotic magnesium chloride. Preparations were superfused with ASW (in mM: 460 NaCl, 10 KCl, 55 MgCl_2_, 11 CaCl_2_, and 10 HEPES buffer, pH 7.6) at 0.3 mL/minute at 14–16 °C. Sharp microelectrodes (3–10 MOhm) were pulled using a P-97 puller (Sutter Instruments), and filled with 0.6 M K_2_SO_4_ and 60 mM KCl electrolyte solution. Membrane potentials and currents were measured with AxoClamp 2B amplifiers (Molecular Devices; San Jose, CA), low-pass filtered to 2 kHz (Frequency Devices; Ottawa, IL), and digitized at 5 kHz with a 1322 A Digidata (Molecular Devices). Voltage clamp recordings were performed with a two-electrode configuration (TEVC). Voltage clamp measurements of currents were made by applying a series of voltage steps, ranging from −90 mV to −20 mV in 10 mV increments. Individual steps were separated by 30–45 seconds, and series of steps were repeated every 5 minutes. Steady state currents were computed by averaging the current over the last 100 milliseconds of each step. To limit space clamp problems, buccal nerve 3, which contains the B48 axon, was either cut or pinched in the neuropil to restrict its extent. Cells with membrane potentials more depolarized than −50 mV were not used. Extracellular recordings were obtained using polyethylene suction electrodes connected to a Model 1700 differential AC amplifier (bandpass 0.1–1 kHz) (A-M Systems; San Diego, CA).

### Analysis of motor programs

Motor programs were triggered by stimulating the command like neuron, Cerebral Buccal Interneuron 2 (CBI-2) at 9–10 Hz, using an established protocol^39,40^. The protraction phase of the motor program was monitored by recording from the I2 nerve. The B48 firing frequency during protraction was calculated as the number of B48 spikes during protraction divided by the protraction duration.

### PKI Experiments

The peptide inhibitor of PKA, PKI 6–22amide (Sigma P6062; Sigma Aldrich, St. Louis, MO) was dissolved at 2 mg/ml in vehicle (250 mM K2S04, 0.2% Fast Green FCF, and 20 mM KHEPES, pH 7.4). Bilateral pairs of B48 neurons were compared, one B48 served as a control, and the other was injected with PKI. In CBI-2 experiments (Fig. 1), a single CBI-2 cell was activated. Across preparations, we alternated whether the B48 ipsilateral to CBI-2 was injected with PKI, or served as the control. Injections of PKI and vehicle were performed at the same time using two picospritzers (Picospritzer II; General Valve, Pine Brook, NJ). Injection pulses were 2–20 msec in duration, between 10–40 psi, and were repeated at a frequency of 0.5 Hz for ~10 minutes. The progression of intracellular loading was monitored by watching the movement of the Fast Green dye. After loading we allowed 5–30 minutes for diffusion to occur. In FCAP + CP2 experiments (Fig. 2) bi-lateral pairs of B48 neurons were loaded with either PKI or vehicle and were impaled with a second micropipette, which was used for the TEVC. Cells were held at −50 mV, and voltage clamp steps applied. After verifying a good space clamp, sea water with 20 µM TTX in ASW (TTX-SW) was washed in for 10 minutes. After a stable baseline was established in TTX, FCAP and CP2 (1 µM each, also in TTX-SW) were superfused onto the preparation for a total of 10 minutes. The resulting change in B48 current was measured 5 minutes after the superfusion began.

### Time course of currents and excitability

A within animal design was used to compare the bilaterally symmetrical B48 neurons. For technical reasons, the excitability of the B48 ipsilateral to CBI-2 was measured in the current clamp configuration, and changes in currents were measured from the B48 contralateral to CBI-2 in the TEVC configuration. This design was necessary since voltage clamping and axotomizing the ipsilateral B48 would damage the CBI-2 axon. Excitability and current measurement were made once every 5 minutes. Excitability was determined by counting the number of spikes evoked by a 3 sec, 1 nA constant current pulse. Currents were measured by applying voltage clamp steps. The holding potential was −60 mV. The two B48 neurons are electrically coupled to each other with an 8% coupling coefficient^16^. To minimize an interaction between the excitability test and the voltage steps, excitability tests preceded voltage steps by 5 sec.

### Measurement of peptide currents during ionic substitution

Buccal ganglia were hemisected, and B48 was axotomized. A single B48 was voltage clamped at −60 mV and voltage clamp steps were applied. For 95% sodium or calcium substitution experiments, steps ranged from −100 to −20 mV in increments of 20 mV. For 50% sodium substitution experiments, steps ranged from −90 to −20 in 10 mV increments. For each cell, after a stable baseline was established in ASW, modified ASW or control ASW was then superfused for 20–30 minutes, and voltage steps were applied again until preparations were stable. Next, FCAP + CP2 (1 µM each) was prepared in either modified ASW or control ASW, and superfused for 10 minutes. Difference (i.e., peptide-induced) currents were determined by subtracting currents recorded before peptides from those recorded during the final 5 minutes of peptide superfusion. Note that no TTX was used in this experiment, and the fast inward current was clipped in plotting the traces to allow visualization of changes to steady state currents. Direct t-tests comparisons were used to test for differences between peptide currents in normal seawater and currents measured in 95% NMDG or cobalt. To examine the effect of substituting 50% of sodium with NMDG, we divided the control current by a factor of 2, and compared this arithmetically computed value to the current measured in 50% sodium. We note that the division of the control current will also scale its variance by half. This reduced variance could increase the likelihood of making a type 1 error. However, this does not apply here, as we did not observe a significant difference. In these experiments, control and experimental currents could not be measured simultaneously. The order of the experiment (i.e. control first or modified ASW first) was alternated to minimize any confound presented by timing.

### Ionotophoresis of cAMP and characterization of cAMP induced currents

cAMP was ionophoresed from double-barreled electrodes. One barrel was filled with 200 mM cAMP (sodium or potassium, pH ~7.3–7.5), and the second barrel with standard filling solution. Iontophoretic injections were 2–5 sec in duration, and 40–60 nA in amplitude. An equal amplitude opposite sign current was passed through the second barrel, so that the membrane potential of the neuron would not change during iontophoresis. To examine the ionic dependence of the cAMP current, responses to iontophoresis were measured once every 2 minutes. After responses stabilized, we changed the bathing solution (i.e., applied the experimental solution). Preparations were maintained in the experimental solution for 10 minutes, and then returned to nASW. To test whether 50% sodium substitution reduced the cAMP current by half, we used the same statistical strategy as described above for the measurement of the peptide current in 50% sodium.

To examine the pharmacological properties of cAMP activated currents, cells were loaded with either Rp-cAMPS loaded by iontophoresis through a third electrode (20 mM, −40 nA for 2 minutes) or vehicle. We then measured neurons’ responses to cAMP iontophoresis after loading. Responses to cAMP iontophoresis were observed before loading, and in control cells. There was no significant effect of the loading control (pre vs. post (t_(2)_ = 0.36, P = 0.75)).

### Loading of Rp-cAMPS after priming

To examine whether the inward current observed following repeated CBI-2 stimulation could be blocked by Rp-cAMPS, an unpaired design was used. The B48 neuron contralateral to CBI-2 was axotomized and voltage clamped at −60 mV. Currents were measured with voltage steps ranging from −80 to −20 mV in 10 mV increments (Fig. 4A). Once a stable baseline was established, ten cycles of motor program activity were elicited by CBI-2 stimulation. One to two minutes later the same series of voltage steps was applied. Next, a third electrode was introduced into each B48, and the cell was iontophoretically loaded with either Rp-cAMPS (20 mM −40 nA for 2 minutes) or vehicle. The third electrode was then removed, and the series of voltage steps was repeated 12 minutes after CBI-2 stimulation. Note in voltage clamp steps to −30 and above, a fast inward current is present, this has been clipped in the traces to allow visualization of steady-state changes.

To determine the effect of Rp on priming in B48 we utilized a similar paradigm with two modifications. First, rather than applying voltage clamp steps 12 minutes after priming we induced a single motor program (Fig. 4F). Second, the B48 examined was maintained in current clamp mode, and only the B48 ipsilateral to CBI-2 was manipulated (either as a control or for Rp loading). We determined the average firing frequency of B48 during the protraction phase of the first cycle of activity triggered during priming (rested), the last cycle triggered during priming (primed), and the final cycle after loading (post priming). As an internal validation of the preparation ‘state’, the activity of B48 contralateral to CBI-2 was monitored. No significant differences were observed in the firing of the contralateral B48 in the post-priming program between preparations where the ipsilateral cell was loaded with Rp as compared to preparations where the ipsilateral B48 was vehicle loaded (t_(8)_ = 1.46, P = 0.18).

### Statistical analysis

Statistical tests were performed using R. For within animal paired comparisons with repeated measures, a repeated measure ANOVA was used, and blocked with each preparation as an error term. For across animal comparisons with repeated measures, an unblocked repeated measures ANOVA was used. Post hoc Student’s T tests were corrected by the Holm-Sidak method. To quantify the persistence of CBI-2 effects on B48 excitability and current induction, we noted the time at which the potentiation of excitability or the inward current fell to less than 37% of its peak value. We used this value as an estimate of the duration of the effect, since the decay of these effects was not well fit by the same function across every preparation. Data presented in summary figures are group means, error bars indicate standard error of the mean. Asterisks in figures indicate significance, (* P < 0.05, ** P < 0.01, *** P < 0.001). P < 0.05 was selected for significance tests.

## Supplementary information

Positive control for PKA inhibition and additional current characterization
