# Supplementary material for: Persistent effects of cyclic adenosine monophosphate are directly responsible for maintaining a neural network state
Source: Sci Rep. 2019 Jun 21;9:9058. doi: 10.1038/s41598-019-45241-9 (PMC6588548; doi:10.1038/s41598-019-45241-9)
Supplement: Supplementary file 1 — Positive control for PKA inhibition and additional current characterization [file 41598_2019_45241_MOESM1_ESM.pdf]

Supplement to: Persistent effects of cyclic adenosine monophosphate are directly responsible for maintaining a neural network state

Perkins, Matthew H.
matthew.perkns@mssm.edu

Weiss, Klaudiusz R.
klaudiusz.weiss@mssm.edu

Cropper, Elizabeth C.
elizabeth.cropper@mssm.edu

April 7, 2019

Supplemental Figures

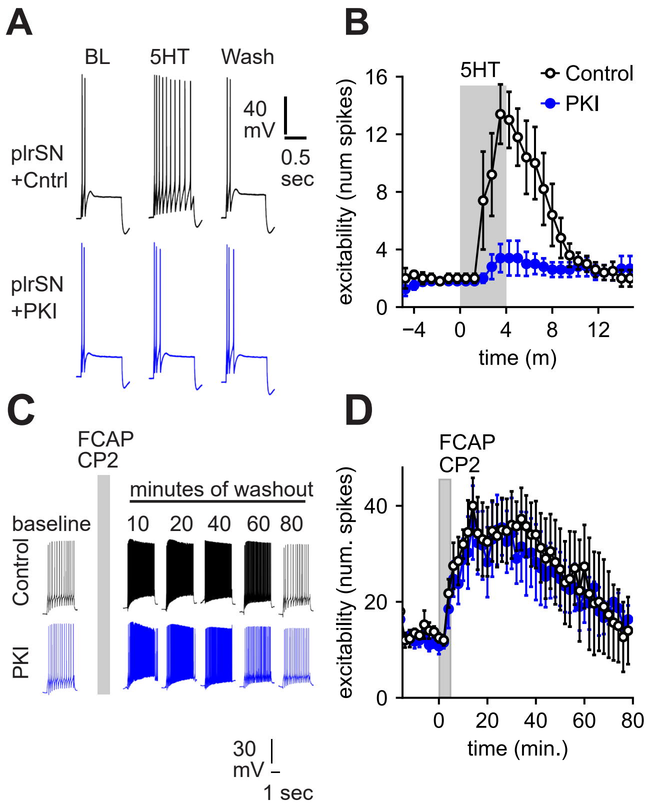

Figure 1: PKI does not block peptide-induced increases in excitability in B48 but does block serotonin (5-HT)-induced increases in excitability in Aplysia pleural sensory neurons (related to Figure 1). (A and B) Sensory neuron excitability was measured by injecting a constant current pulse before 5-HT (baseline (BL)), in the presence of 20 μM 5-HT (5HT), and for 10 min after 5-HT washout in neurons preloaded with vehicle (Control, black) and in neighboring neurons preloaded with 2 mM PKI (PKI, blue). PKI loading blocked 5-HT induced excitability increases. (C and D) Peptide induced increases in B48 excitability persist in the presence of PKI. B48 excitability was measured by injecting a constant current pulse before peptides (baseline) and for 80 min after peptides in pairs of neurons. In each pair, one B48 was injected with vehicle (control, black) and the opposite-side B48 was injected with PKI (blue). The gray bars indicate peptide superfusion (FCAP CP2 1μM each). PKI loading had no effect ($F_{(1,263)}$=3.24, P =0.073, N=4). The time it took for the peptide effect to fall to 37% of its peak, was 58.0±6.98 min for vehicle loaded cells, and 70.5±7.5 minutes for PKI loaded cells ($t_{(3)}$=1.05, P=0.37, N=4). Sample sizes: Panel B (N=5), Panel D (N=4), where N = number of preparations.

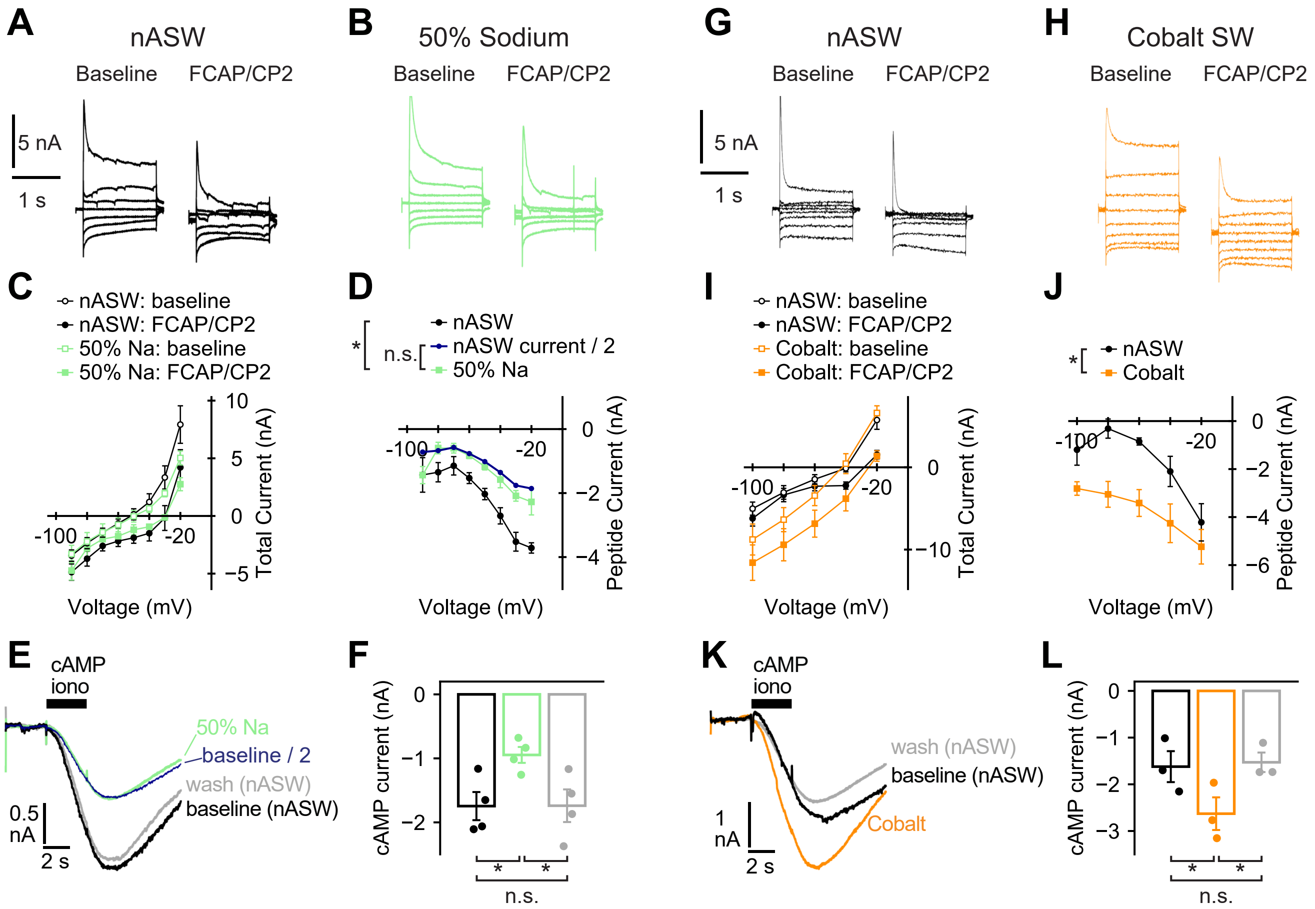

Figure 2: Currents induced by peptides and direct application of cAMP are similar (related to Figure 3). (A-F) Sodium is the primary charge carrier of both the peptide-induced current, and the cAMP-activated current. In (A)-(D) peptide-induced currents were measured during 2 s steps delivered between -90 and -20 mV in 10 mV increments from a holding potential of -60 mV. Recordings were made before (baseline) and after peptide superfusion (FCAP CP2 1μM each) in nASW (nASW, black) and in a saline with 50% of the sodium removed (50% Sodium, green). Total currents for all four conditions are plotted in (C). In (D) difference (i.e., peptide-induced) currents are plotted in nASW (black) and in 50% Na (green). For comparison, currents in nASW divided by two are also plotted (dark blue). Note that reducing the extracellular sodium by 50% produced a proportional decrease in the current. Taken together with data shown in Figures 3A and 3B this indicates that the peptide induced current is primarily a sodium current. In (E and F) currents were induced by cAMP iontophoresis at -60 mV (black bar labeled cAMP iono) in nASW (baseline, black), in a saline with 50% of the sodium removed (50% Na, green), and after returning to nASW (wash, gray). In (E) the current measured in nASW divided by two is also plotted for comparison (dark blue). In (E) each trace is an average of 5-6 individual responses. Note that reducing the extracellular sodium by 50% produced a proportional decrease in the cAMP induced current. Taken together with data shown in Figures 3C and 3D this indicates that the cAMP induced current is primarily a sodium current. (G-L) Peptide and cAMP induced currents are both potentiated in low calcium saline. In (G)-(J) peptide-induced currents were measured during 2 s steps delivered between -100 and -20 mV in 20 mV increments from a holding potential of -60 mV. Recordings were made before (baseline) and after peptide superfusion (FCAP CP2 1μM each) in nASW (nASW, black) and in a saline in which cobalt substituted for calcium (Cobalt, orange). Total currents for all four conditions are plotted in (I). In (J) difference (i.e., peptide-induced) currents are plotted in nASW (black) and in cobalt saline (orange). Note that reducing the extracellular calcium increased the amount of current induced by peptide application. In (K and L) currents were induced by cAMP iontophoresis at -60 mV (black bar labeled cAMP ino) in nASW (baseline, black), in a saline in which cobalt substituted for calcium (cobalt, orange), and after returning to nASW (wash, gray). In (K) each trace is an average of 5-6 individual responses. Note that reducing the extracellular calcium potentiated the cAMP induced current, as it did the peptide-induced current. Sample sizes: Panels C and D (N=6), Panel F (N=4), Panels I and J (N=3), Panel L (N=3)
